# The competing mini-dumbbell mechanism: new insights into CCTG repeat expansion

**DOI:** 10.1038/sigtrans.2016.28

**Published:** 2016-12-02

**Authors:** Pei Guo, Sik Lok Lam

**Affiliations:** 1 Department of Chemistry, The Chinese University of Hong Kong, Shatin, Hong Kong

## Abstract

CCTG repeat expansions in intron 1 of the cellular nucleic acid-binding protein gene are associated with myotonic dystrophy type 2. Recently, we have reported a novel mini-dumbbell (MDB) structure formed by two CCTG or TTTA repeats, which potentially has a critical role in repeat expansions. Here we present a mechanism, called the competing MDB mechanism, to explain how the formation of MDB can lead to efficient mismatch repair (MMR) escape and thus CCTG repeat expansions during DNA replication. In a long tract of CCTG repeats, two competing MDBs can be formed in any segment of three repeats. Fast exchange between these MDBs will make the commonly occupied repeat behave like a mini-loop. Further participations of the 5′- or 3′-flanking repeat in forming competing MDBs will make the mini-loop shift in the 5′- or 3′-direction, thereby providing a pathway for the mini-loop to escape from MMR. To avoid the complications due to the formation of hairpin conformers in longer CCTG repeats, we made use of TTTA repeats as model sequences to demonstrate the formation of competing MDBs and shifting of mini-loop in a long tract of repeating sequence.

## Introduction

In the human genome, expansions of CCTG repeats found in intron 1 of the cellular nucleic acid-binding protein (*CNBP*) gene on chromosome 3q21 are associated with a complex multisystem disorder called myotonic dystrophy type 2 (DM2).^1^ These repeats are located in a part of the complex motif (TG)_14–25_(TCTG)_4–10_(CCTG)*_n_* (where *n* is the repeat length).^1–3^ In normal individuals, *n* is usually below 30 and the CCTG repeat tract is interrupted by one or more A/G/TCTG motif(s). In DM2 patients, *n* can vary between 55 and ~11 000 and the expansions of CCTG repeats are extremely variable.^4^ At present, the mechanism of CCTG repeat expansions remains elusive.

For repeat expansions to occur, one commonly accepted pathway involves the formation of an unusual structure in the nascent strand during DNA replication.^5–7^ Meanwhile, the unusual structure can also form during DNA repair or recombination.^8–13^ In general, the unusual structure will be recognized and removed by mismatch repair (MMR), which is a post-replication repair system to maintain the fidelity of DNA replication. MMR proteins will search for structural artefacts such as non-Watson–Crick base pairs or unpaired nucleotides and then excise them. As a result, both the formation of unusual structure and repair escape must occur to bring about repeat expansions. For CCTG repeats, it has been shown that they can adopt a variety of different unusual structures including hairpin, dumbbell and mini-dumbbell (MDB).^14–16^ Yet it remains unclear how these unusual structures escape from MMR. In this study, we present a novel mechanism, called the competing MDB mechanism, to explain how efficient MMR escape can occur via the formation of MDBs.

**Figure 1:**
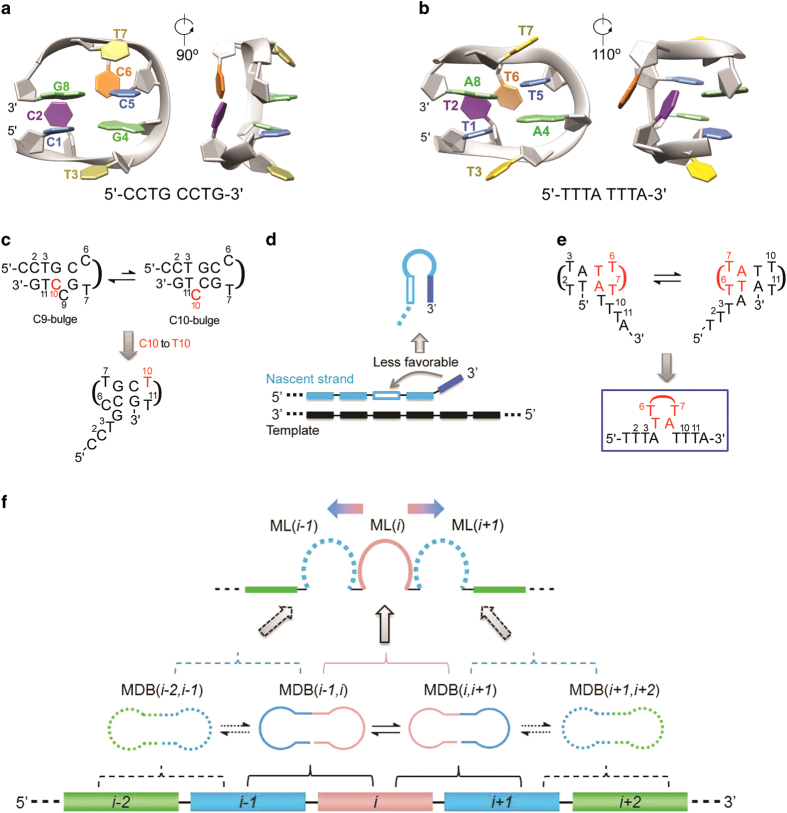
The averaged structures of (**a**) CCTG MDB (PDB ID: 5GWL) and (**b**) TTTA MDB (PDB ID: 5GWQ). The two minor groove residues C2 and C6 form a mispair in CCTG MDB whereas T2 and T6 stack with each other in TTTA MDB. (**c**) Hairpins were formed in a sequence containing three CCTG repeats. Through single-site substitution experiment to weaken the stabilizing interactions between the first and third repeats, an MDB with a 5′-overhang was formed. (**d**) During DNA replication, end-fray occurs mainly at the 3′-end of the nascent strand and the 5′-end is hybridized with the template. Therefore, the 3′-terminal repeat cannot interact with its preceding repeats feasibly, thus hindering the formation of hairpin. (**e**) Two types of MDBs were formed in a sequence containing three TTTA repeats. Fast exchange between them made the sequence behave like a mini-loop. (**f**) A schematic diagram of the competing MDB mechanism. The mini-loop can shift in both the 5′- and 3′-directions. ML(*i*) cannot coexist with either ML(*i*−1) or ML(*i*+1).

For the reported MDB formed by two CCTG repeats, it comprises of a 5′- and a 3′-type II loops.^15,16^ A type II loop is defined by its distinctive folding geometry in which the first and fourth loop residues form the loop-closing base pair, the second loop residue is positioned in the minor groove, whereas the third loop residue stacks on the loop-closing base pair.^17^ In the 5′- and 3′-loops of this MDB, C1 and G4, and C5 and G8 form the two loop-closing base pairs (Figure 1a). C2 and C6 fold into the minor groove and form a mispair, whereas T3 and T7 stack on C1-G4 and C5-G8, respectively. Apart from the pairing geometry of the minor groove residues, the structural features of CCTG MDB are similar to those of another MDB formed by two TTTA repeats (Figure 1b).^15,18^ Instead of forming a mispair, the minor groove residues T2 and T6 of TTTA MDB stack with each other. In these two MDBs, there are multiple stabilizing loop-loop interactions which are absent in larger dumbbell structures.^14,19,20^ To understand how potentially CCTG MDB participates in MMR escape, we first consider the dynamic processes that have been observed in a DNA tract containing three CCTG repeats.^14^ Instead of forming any MDB, two hairpin conformers were formed due to the stabilizing interactions between the first and third repeats (Figure 1c, top). During DNA replication, these interactions are expected to be less favorable in the nascent strand containing CCTG repeats because the 5′-end is hybridized with the template and end-fray occurs mainly at the 3′-end (Figure 1d). Through single-site substitution experiment to weaken these interactions between the first and third repeats, we demonstrated that an MDB could be formed (Figure 1c, bottom).^16^ Thereby, in a segment of three repeats in a longer CCTG tract, there can be the formation of two possible types of MDBs, one containing a 5′-overhanging repeat and the other containing a 3′-overhanging repeat. Interestingly, these MDBs have been found in a DNA sequence containing three TTTA repeats and fast exchange between them makes the sequence behave like a mini-loop as evidenced by the unusually shifted ^31^P signals of the second repeat at 25 °C (Figure 1e).^18^ At lower temperatures, all ^1^H and ^31^P peaks were broadened, suggesting the presence of conformational exchange between two competing MDBs as this process involves the unfolding and refolding of the first and third repeats. If an hairpin was formed in this sequence, these signals would remain sharp due to a further stabilization of the hairpin at lower temperatures. Because there is no complication due to the formation of hairpin in TTTA repeats, here we employed longer sequences of TTTA repeats to serve as models to rationalize how CCTG repeat expansions occur via the competing MDB mechanism.

To elaborate the competing MDB mechanism, we first explain the concept of competing MDBs with a longer tract of repeats. In this longer tract, every two adjacent repeats are capable of forming an MDB (Figure 1f). For instances, the folding of the (*i*−1)th and *i*th repeats results in the formation of MDB(*i*−1,*i*), and the folding of the *i*th and (*i*+1)th repeats results in the formation of MDB(*i*,*i*+1). If the formation of two MDBs requires the use of a common repeat, for example, both MDB(*i*−1,*i*) and MDB(*i*,*i*+1) require the use of *i*th repeat, these two MDBs will compete with each other and we call them the competing MDBs. Fast exchange between these competing MDBs will make the *i*th repeat behave like a mini-loop, ML(*i*). If the 5′- or 3′-flanking repeat of these competing MDBs also participates in the formation of MDB(*i*−2,*i*−1) or MDB(*i*+1,*i*+2), then fast exchange between (i) MDB(*i*−2,*i*−1) and MDB(*i*−1,*i*), and (ii) MDB(*i*,*i*+1) and MDB(*i*+1,*i*+2) will result in the formation of ML(*i*−1) and ML(*i*+1), respectively. Thereby, a shifting of the mini-loop from the *i*th to (*i*−1)th or (*i*+1)th repeat will occur and this bi-directional shifting of the mini-loop forms the basis of the competing MDB mechanism.

## Materials and methods

### DNA samples

The sequences containing four to eight TTTA repeats were investigated in this study, and they were named as ‘(TTTA)_4–8_’. To investigate the effects of 5′- and 3′-flanking residues on the formation of MDB, we also designed two other sequences by adding an adenine residue to the 5′-terminal and a thymine residue to the 3′-terminal of two TTTA repeats, respectively. These sequences were named as ‘A(TTTA)_2_’ and ‘(TTTA)_2_T’. All DNA samples were synthesized using an Applied Biosystems model 394 DNA synthesizer (Applied Biosystems, Foster City, CA, USA). They were purified by denaturing polyacrylamide gel electrophoresis and diethylaminoethyl Sephacel anion exchange column chromatography, and finally desalted using Amicon Ultra-4 centrifugal filtering devices (Millipore Corporation, Billerica, MA, USA). Nuclear magnetic resonance (NMR) samples were prepared by dissolving 0.3 μmol purified DNA into 500 μl buffer solutions containing 10 mm sodium phosphate (pH 7.0), and 0.1 mm 2,2-dimethyl-2-silapentane-5-sulfonic acid.

### NMR study

All NMR experiments were performed using Bruker AV-500 and/or AV-700 spectrometers (Bruker BioSpin AG, Faellanden, Switzerland). For studying the labile protons, the samples were prepared in a 90% H_2_O/10% D_2_O buffer solution. One-dimensional imino and two-dimensional (2D) nuclear Overhauser effect spectroscopy experiments were performed using excitation sculpting to suppress the water signal.^21^ For studying the non-labile protons, the solvent was exchanged with a 99.96% D_2_O solution and a 2-s presaturation pulse was used to suppress the residual water signal. 2D nuclear Overhauser effect spectroscopy spectra were acquired with a data size of 4096×512 and a mixing time of 300 ms unless otherwise specified. The acquired data sets were zero-filled to give 4096×4096 spectra with a cosine window function applied to both dimensions. Backbone ^31^P signals were assigned using 2D total correlation spectroscopy with a mixing time of 75 ms and ^1^H–^31^P heteronuclear single-quantum coherence experiments. The ^31^P spectral width was set to 6 p.p.m. and a data size of 4096×200 was collected. The ^31^P chemical shifts were indirectly referenced to 2,2-dimethyl-2-silapentane-5-sulfonic acid using the derived nucleus-specific ratio of 0.404808636.^22^

### Native gel assay

Native gels composed of 25% polyacrylamide were prepared to investigate the oligomeric states of (TTTA)_4–8_. The gel loading samples were prepared in the same NMR buffer solution. For the reference lane, a DNA ladder composed of 10–100-nt single-strand oligomers was used. The polyacrylamide gel electrophoresis experiments were conducted at both ~25 and ~5 °C. To maintain the pH at 7.0, an electrophoretic buffer containing 9 mm piperazine-*N*,*N*′-bis(2-ethanesulfonic acid), 20 mm bis(2-hydroxyethyl)-amino-tris(hydroxymethyl)-methane and 1 mm ethylenediaminetetraacetic acid was used. DNA bands were visualized by post-staining the gels with stains-all solution.

## Results and Discussion

Sequential assignments of (TTTA)_4–8_, A(TTTA)_2_ and (TTTA)_2_T were made from the 2D nuclear Overhauser effect spectroscopy H6/H8-H1′ fingerprint regions using standard methods^23,24^ and the results are shown in Supplementary Figures S1–S4. Based on the H3′ assignments from total correlation spectroscopy spectra, the ^31^P assignments of (TTTA)_4–8_, A(TTTA)_2_ and (TTTA)_2_T were made using ^1^H–^31^P heteronuclear single-quantum coherence spectra and the results are shown in Supplementary Figures S5–S8.

### (TTTA)_4_ behaves like a mini-loop at the third repeat

**Figure 2:**
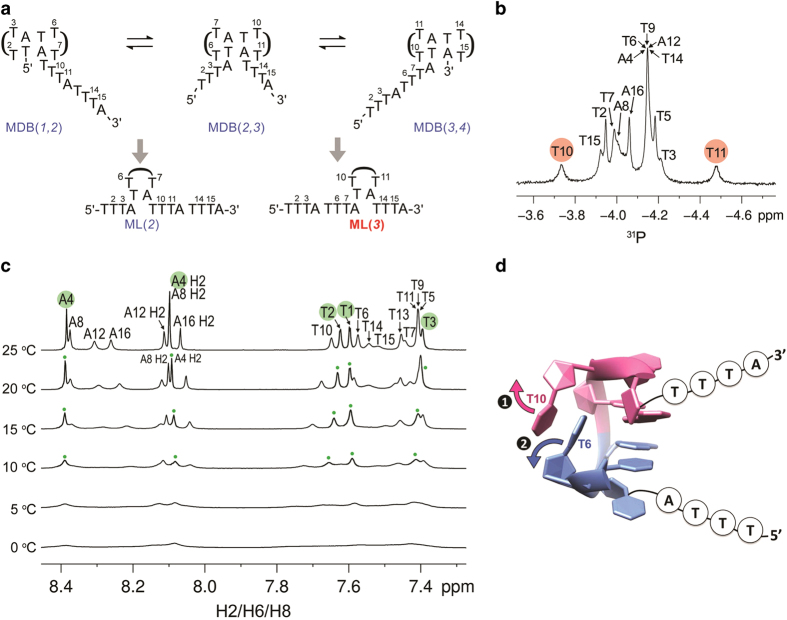
(**a**) Three types of MDBs can be formed in (TTTA)_4_ (top). Owing to steric clashes, the formation of MDB(1,2) disfavors the formation of MDB(3,4) and vice versa. Fast exchange between MDB(1,2) and MDB(2,3), and MDB(2,3) and MDB(3,4) results in ML(2) and ML(3), respectively. (**b**) At 25 °C, the unusually downfield T10 and upfield T11 ^31^P signals suggest (TTTA)_4_ behaves like ML(3) with a rapid exchange between MDB(2,3) and MDB(3,4). (**c**) Peak broadenings were observed mainly from the proton signals belonging to the last three repeats. For easy identification, the sharper peaks from the first repeat were labeled in green. At lower temperatures, all proton signals were broadened, suggesting all four repeats were involved in conformational exchange processes. (**d**) Owing to T6 being sandwiched between T10 and the loop-closing base pairs, the 3′-loop unfolds before the 5′-loop in MDB(2,3), making the formation of MDB(3,4) occur before that of MDB(1,2).

In a DNA strand containing four TTTA repeats, three types of MDBs can be formed using the first two, the middle two and the last two repeats, respectively. These result in MDB(1,2) with a two-repeat 3′-overhang, MDB(2,3) with one repeat in each of the overhangs, and MDB(3,4) with a two-repeat 5′-overhang (Figure 2a). Based on the competing MDB mechanism, fast exchange between MDB(1,2) and MDB(2,3) makes the sequence behave like a mini-loop at the second repeat, ML(2). Similarly, fast exchange between MDB(2,3) and MDB(3,4) results in a mini-loop at the third repeat, ML(3). If ML(2) and ML(3) underwent slow exchange, the NMR spectral features of these two mini-loops would appear. If there were fast exchange between ML(2) and ML(3), there would be a loss of the mini-loop spectral features. Surprisingly, we observed only the NMR spectral features of ML(3) at 25 °C, for example, the unusual ^31^P chemical shifts of T10 and T11 (Figure 2b), which are the NMR characteristics of type II TTTA loop.^18,25^ As supported by the fact that most of the proton signals from the last three repeats were relatively more broadened than those from the first repeat (Figure 2c), competing MDBs seem to form more preferably in the last three repeats.

To rationalize the appearance of only ML(3) at 25 °C, we made use of the competing MDB mechanism. In (TTTA)_4_, ML(2) comes from the fast exchange between MDB(1,2) and MDB(2,3), whereas ML(3) comes from the fast exchange between MDB(2,3) and MDB(3,4). As the formation of both mini-loops involves the exchange with MDB(2,3), therefore the unfolding of MDB(2,3) has a critical role in these exchange processes. In the detailed TTTA MDB structure (Figure 1b), the minor groove residue T2 is sandwiched between the loop-closing base pairs and T6, making T2 being better stabilized in the minor groove than T6. Therefore, the unfolding of the 3′-loop (third repeat) is expected to occur before that of the 5′-loop (second repeat) in MDB(2,3) (Figure 2d). This will make the unfolded third repeat interact with the last repeat to form MDB(3,4) before the second repeat interacts with the first repeat to form MDB(1,2). As a result, MDB(2,3) will exchange more efficiently with MDB(3,4) than MDB(1,2), leading to the appearance of only ML(3). Upon lowering the temperature, conformational exchange between MDB(2,3) and MDB(3,4) became slower, promoting also the exchange between MDB(2,3) and MDB(1,2) and thus the formation of ML(2). As evidenced by the peak broadenings of all ^1^H signals at 10 °C and below (Figure 2c), all four repeats were found to participate in the two MDB exchange processes, resulting in both ML(2) and ML(3). The presence of these two mini-loops suggests that the mini-loop can shift in both the 5′- and 3′-directions.

### The presence of flanking repeats drives the unfolding of MDB

From the above analysis, the appearance of ML(3) in (TTTA)_4_ at 25 °C was originated from the prior unfolding of the 3′-loop in MDB(2,3), which then interacted with the 3′-flanking repeat to form MDB(3,4). Upon slowing down the exchange at lower temperatures, the 5′-loop in MDB(2,3) became also available to interact with the 5′-flanking repeat to form MDB(1,2). To better understand the effects from the flanking repeats, we also determined the influences from the 5′- and 3′-nearest neighboring residues of TTTA MDB in this study.

**Figure 3:**
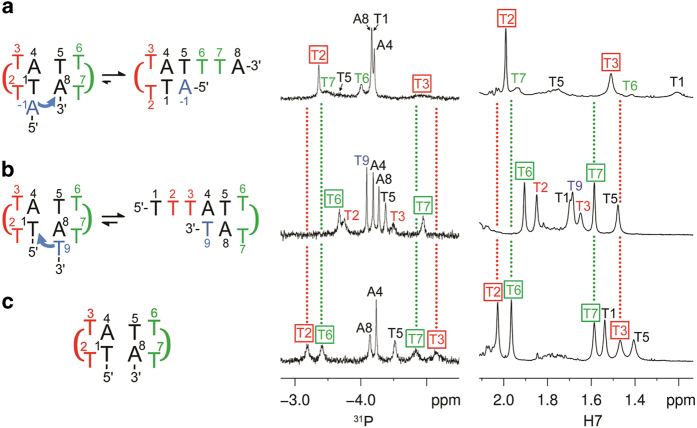
1D ^31^P and ^1^H NMR spectra of (**a**) A(TTTA)_2_, (**b**) (TTTA)_2_T and (**c**) TTTA MDB reference. The ^31^P/H7 chemical shifts of T6 and T7 in A(TTTA)_2_, and T2 and T3 in (TTTA)_2_T are very different from those of TTTA MDB reference, suggesting the 3′-loop of A(TTTA)_2_ and the 5′-loop of (TTTA)_2_T were unfolded, respectively. The 5′-flanking A-1 in A(TTTA)_2_ and the 3′-flanking T9 in (TTTA)_2_T probably compete with A8 and T1 to form base pairs with T5 and A4, respectively. All these spectra were acquired at 10 °C.

For studying the 5′-nearest neighbor effect, we analyzed the NMR spectroscopic results of A(TTTA)_2_ and found that the ^31^P/H7 chemical shifts of T2 and T3 were close to those of TTTA MDB (Figure 3a). However, their T6 and T7 chemical shifts were quite different. These results suggest that the presence of the 5′-flanking A-1 does not affect much on the 5′-loop of TTTA MDB but it leads to the unfolding of the 3′-loop, probably due to A-1 competes with A8 to form a Watson–Crick base pair with T5. For (TTTA)_2_T, which shows the 3′-nearest neighbor effect, the ^31^P/H7 chemical shifts of T6 and T7 remain close to those of TTTA MDB (Figure 3b). However, their T2 and T3 chemical shifts were very different. These suggest that the 3′-flanking T9 has no significant effect on the 3′-loop of TTTA MDB but it probably competes with T1 to form a Watson–Crick base pair with A4, thereby leading to the unfolding of the 5′-loop. The results of A(TTTA)_2_ and (TTTA)_2_T suggest that the presence of 5′- or 3′-flanking residues facilitates the unfolding of MDB.

### Preferential appearance of mini-loop at the 3′-penultimate repeat

**Figure 4:**
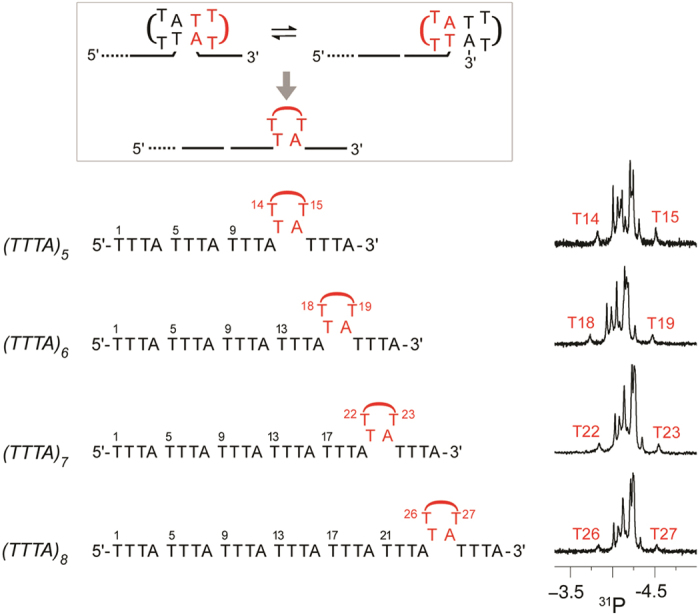
The unusually downfield or upfield ^31^P signals of T14 and T15 in (TTTA)_5_, T18 and T19 in (TTTA)_6_, T22 and T23 in (TTTA)_7_, and T26 and T27 in (TTTA)_8_ suggest a mini-loop appears at the penultimate repeat of these sequences. The mini-loop is resulting from fast exchange between two competing MDBs formed in the last three repeats. The ^31^P spectra shown here were acquired at 25 °C.

To further demonstrate that the competing MDB mechanism provides a pathway for the unusual structure to shift to a different position along a tract of TTTA repeats, we also prepared longer sequences containing five to eight TTTA repeats. Interestingly, all these sequences show a mini-loop at the 3′-penultimate repeat at 25 °C as supported by the unusually downfield or upfield signals of T14 and T15 in (TTTA)_5_, T18 and T19 in (TTTA)_6_, T22 and T23 in (TTTA)_7_, and T26 and T27 in (TTTA)_8_ (Figure 4). The preferential 3′-pemultiamte repeat position can be rationalized by the sequential unfolding of the 3′- and 5′-loops that we encountered in (TTTA)_4_, further consolidating the presence of competing MDBs in these sequences.

Upon lowering the temperature, conformational exchange between the two competing MDBs in the last three repeats became slower, making the formation of competing MDBs towards the 5′-direction more feasible. As supported by the broadenings of all ^31^P signals at lower temperatures (Supplementary Figure S9), all repeats were found to participate in MDB exchange processes. Thereby, the resulting mini-loops are capable to shift in both the 5′- and 3′-directions. To verify that these peak broadenings were not resulting from the formation of multimeric conformers, we also performed native gel analysis. As the mobilities of these repeating sequences were found to be similar to those of single-strand references, these results suggest that all these sequences adopt monomeric conformations at both ~25 and ~5 °C (Supplementary Figure S10).

### Competing MDBs contribute to efficient repair escape

The unusual structure formed in the nascent strand during DNA replication is usually recognized and removed by MMR proteins. Generally, MMR proteins make use of the local weakening due to mismatch/misalignment to locate the unusual structure.^26^ As there is a conserved binding site in MMR proteins for the phosphate of unpaired or mismatched nucleotides,^27–30^ it is expected that local structural changes of the mismatch/misalignment site will affect the recognition by MMR proteins, thus providing a possible pathway for MMR escape. In this study, our results reveal that in a long tract of TTTA repeats, the mini-loop resulting from competing MDBs has a tendency to shift from the 5′- to 3′-direction at 25 °C because there is a sequential unfolding of the 3′- and 5′-loops in TTTA MDB. We believe the translational movement of the mini-loop along the repeat tract will provide a more efficient MMR escape pathway than that resulting from local structural changes. At lower temperatures, shifting of the mini-loop can happen in both the 5′- and 3′-directions due to slower exchange in competing MDBs. This bi-directional movement will further enhance the ability of the mini-loop to escape from MMR.

**Figure 5:**
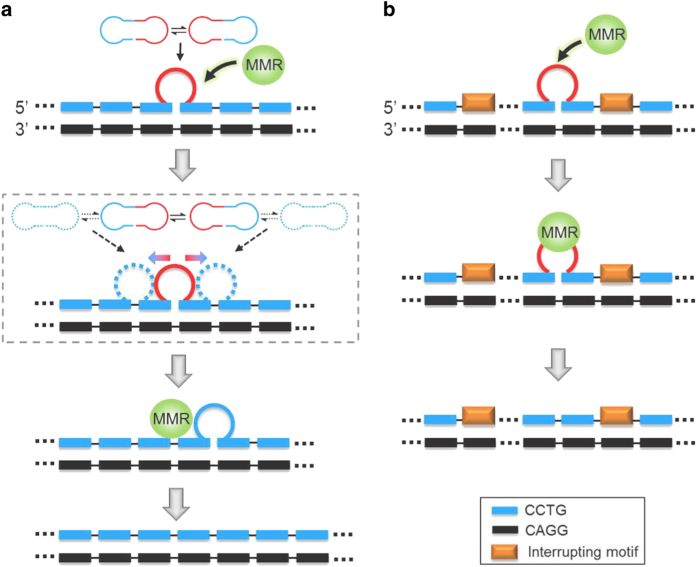
(**a**) In the uninterrupted CCTG repeat tract of DM2 patients, the mini-loop can shift in both directions, resulting in a more efficient escape from MMR. (**b**) In normal individuals, the presence of interrupting motifs in the CCTG repeat tract hinders the shifting of mini-loop. Thereby, the mini-loop can be recognized and removed more efficiently by MMR proteins.

In CCTG MDB, there is no sequential unfolding of the 3′- and 5′-loops because the two minor groove residues do not stack with each other. Instead, they align in the same plane and form a mispair.^15^ As a consequence, the mini-loop resulting from competing CCTG MDBs can shift in both the 5′- and 3′-directions. In the long and uninterrupted CCTG repeat tract of DM2 patients, intrinsically, the mini-loop formed in the nascent CCTG strand has an equal chance to shift towards the 5′- and 3′-directions. During DNA replication, the local environment of the nascent strand will keep changing due to changes in torsional stress resulting from supercoiling induced by replication activities such as the approaching and departure of different proteins.^31^ Therefore, the shifting direction of the mini-loop will also be affected, making the mini-loop shift in an unpredictable direction along the CCTG repeat tract and thus enhancing the capability of the mini-loop to escape from MMR (Figure 5a). In normal individuals, intron 1 of the *CNBP* gene contains only a short CCTG repeat tract that is interrupted by A/G/TCTG motifs. As a consequence, the effect of bi-directional shifting of mini-loop will be limited and thereby the mini-loop can be recognized and removed by MMR more efficiently (Figure 5b).
