# Supplementary material for: The competing mini-dumbbell mechanism: new insights into CCTG repeat expansion
Source: Signal Transduct Target Ther. 2016 Dec 2;1:16028–. doi: 10.1038/sigtrans.2016.28 (PMC5661647; doi:10.1038/sigtrans.2016.28)

The competing mini-dumbbell mechanism: new insights into CCTG repeat expansion

Pei Guo and Sik Lok Lam*

Department of Chemistry, The Chinese University of Hong Kong, Shatin, New Territories, Hong Kong

* Corresponding author. Phone: +852 3943 8126, Fax: +852 2603 5057.

E-mail address: lams@cuhk.edu.hk

Supporting Information (S1-S10)

S1. NOESY H6/H8-H1' fingerprint regions of *(TTTA)$_4$* at a mixing time of **(a)** 300, and **(b)** 800 ms. The sequential NOEs of T10 H1'-T11 H6, T11 H1'-A12 H8, T14 H1'-T15 H6 and T15 H1'-A16 H8 were relatively weak at a mixing of 300 ms. These NOEs became more intense at a mixing of 800 ms. The spectra were acquired at 25 °C.

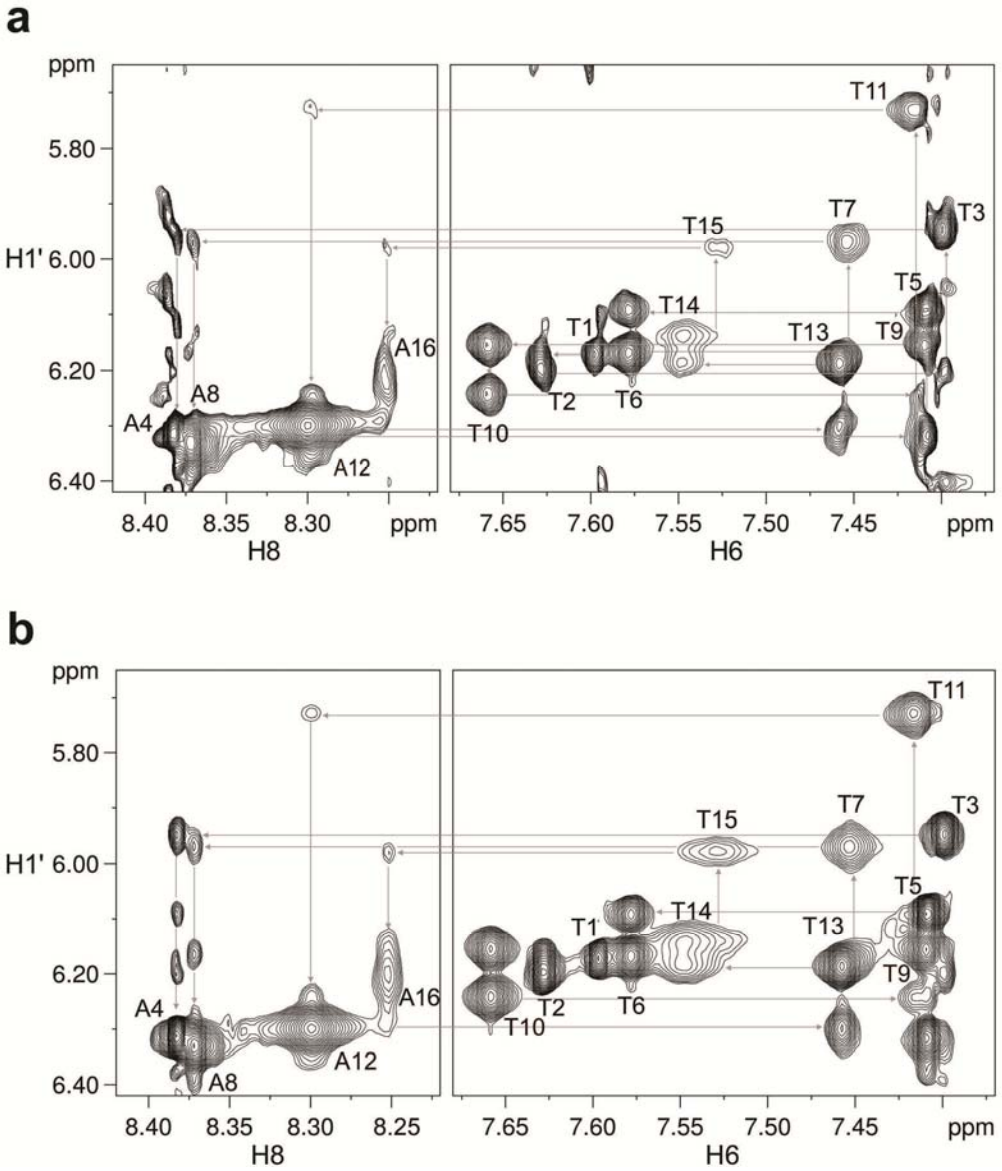

S2. NOESY H6/H8-H1' fingerprint regions of **(a)** *(TTTA)*$_5$ and **(b)** *(TTTA)*$_6$. The NOESY spectra were acquired at 25 °C with a mixing time of 800 ms.

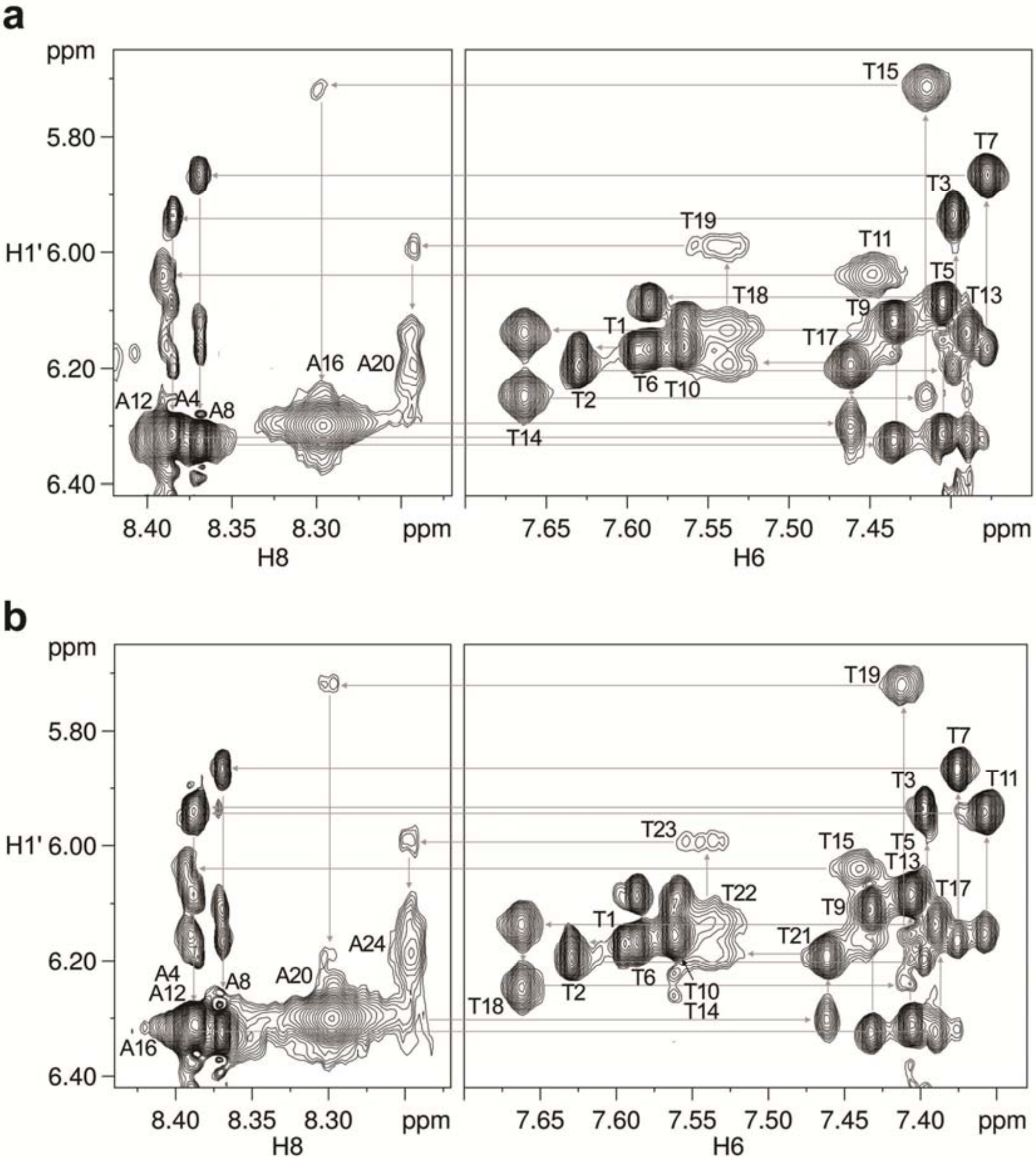

S3. NOESY H6/H8-H1' fingerprint regions of **(a)** *(TTTA)₇* and **(b)** *(TTTA)₈*. Owing to serious peak overlap, the assignments could only be partially completed near the 3'-end as the signals from the 3'-terminal adenine residue were well-resolved. The spectra were acquired at 25 °C with a mixing time of 800 ms.

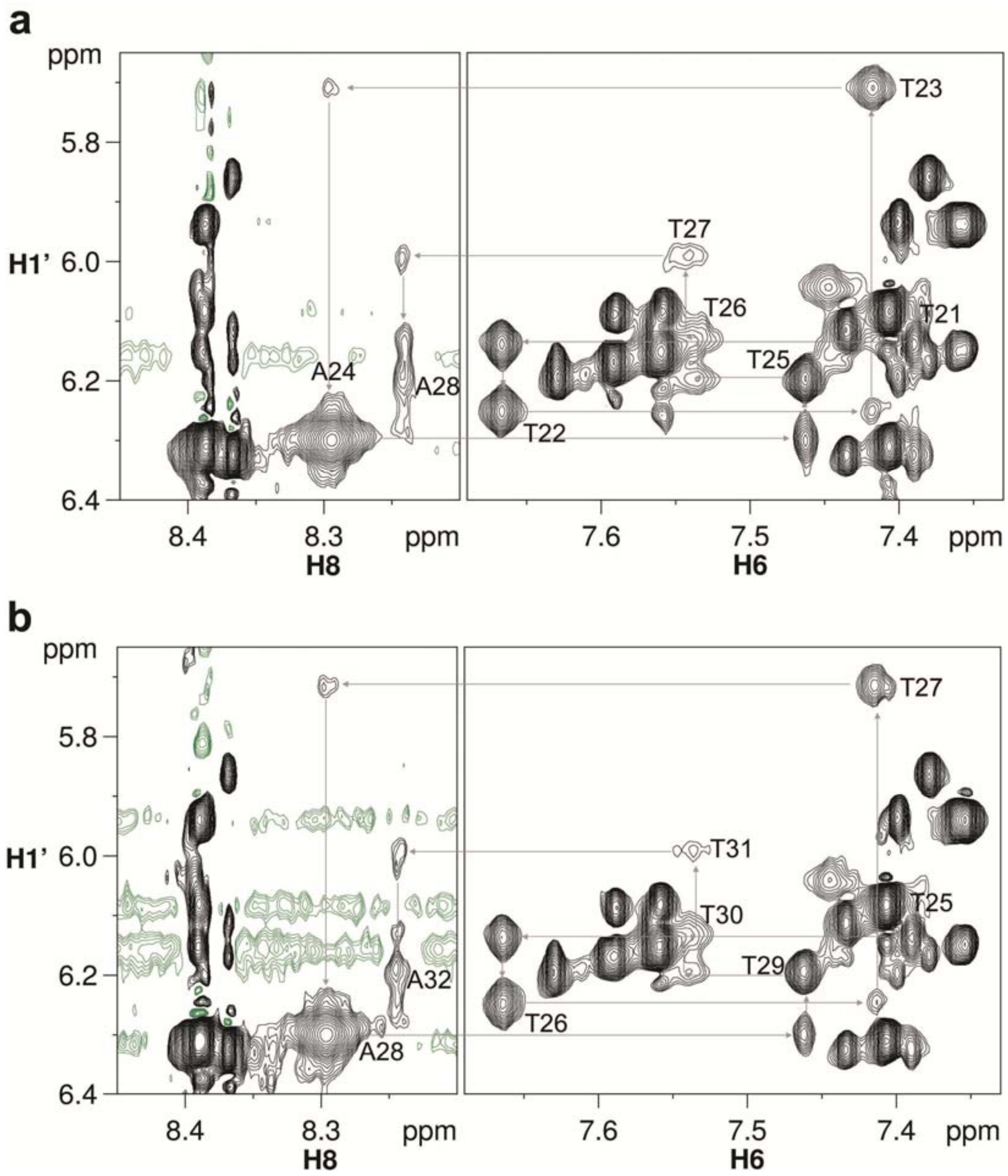

S4. NOESY H6/H8-H1' fingerprint regions of **(a)** *A(TTTA)*$_2$ at 30 °C and **(b)** *(TTTA)*$_2$*T* at 15 °C. The NOE cross peak of A8 H1'-T1 H6 in *(TTTA)*$_2$*T* is highlighted in red, suggesting there is stacking between the 5' and 3' ends in this MDB structure. The spectra were acquired at a mixing time of 800 ms.

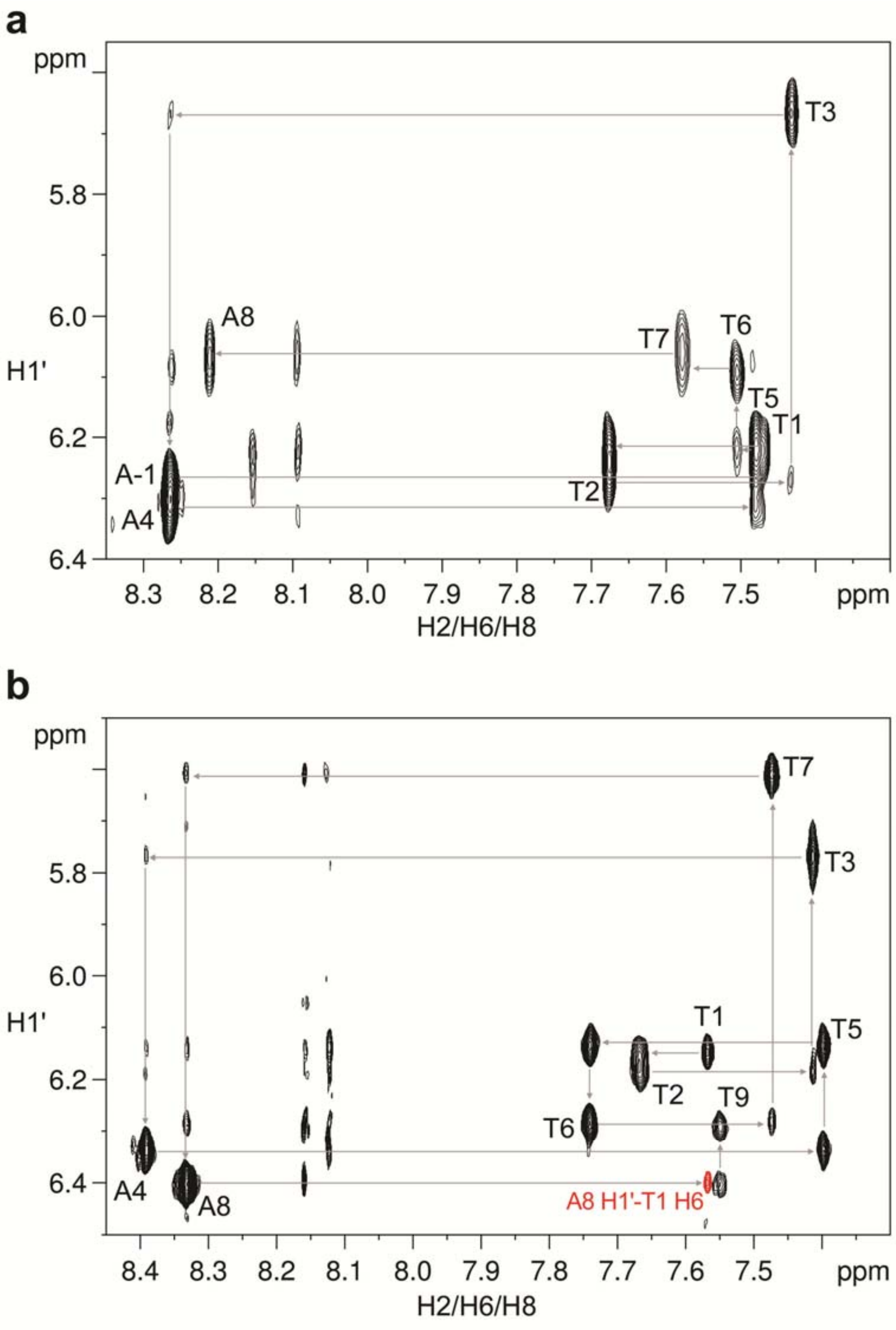

S5. ^{31}P assignment of *(TTTA)₄* using H2'/H2''-H3' and H1'-H3' TOCSY cross peaks and H3'-^{31}P HSQC cross peaks. The spectra were acquired at 25 °C.

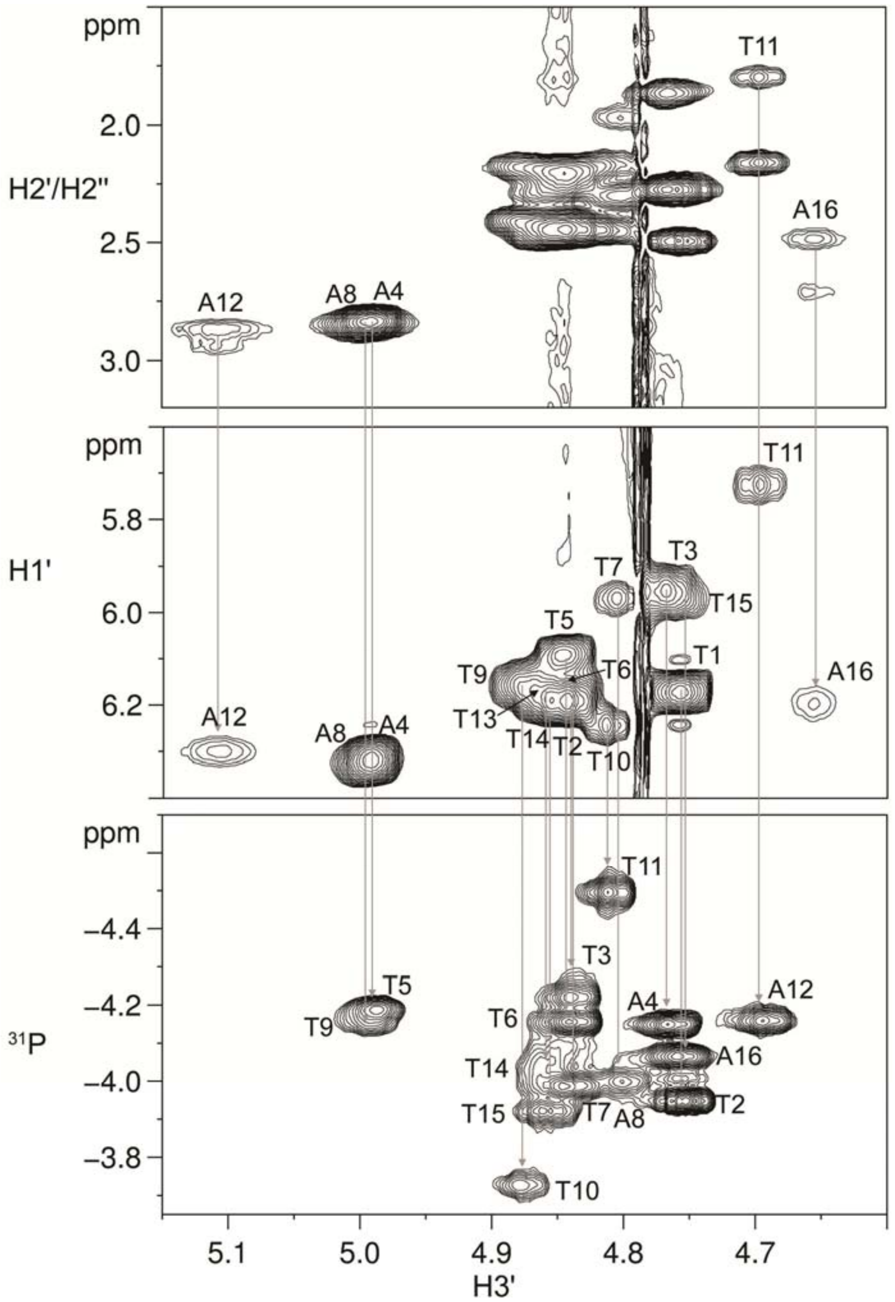

S6. ^{31}P assignment of *(TTTA)$_5$* using H2'/H2''-H3' and H1'-H3' TOCSY cross peaks and H3'-^{31}P HSQC cross peaks. The spectra were acquired at 25 °C.

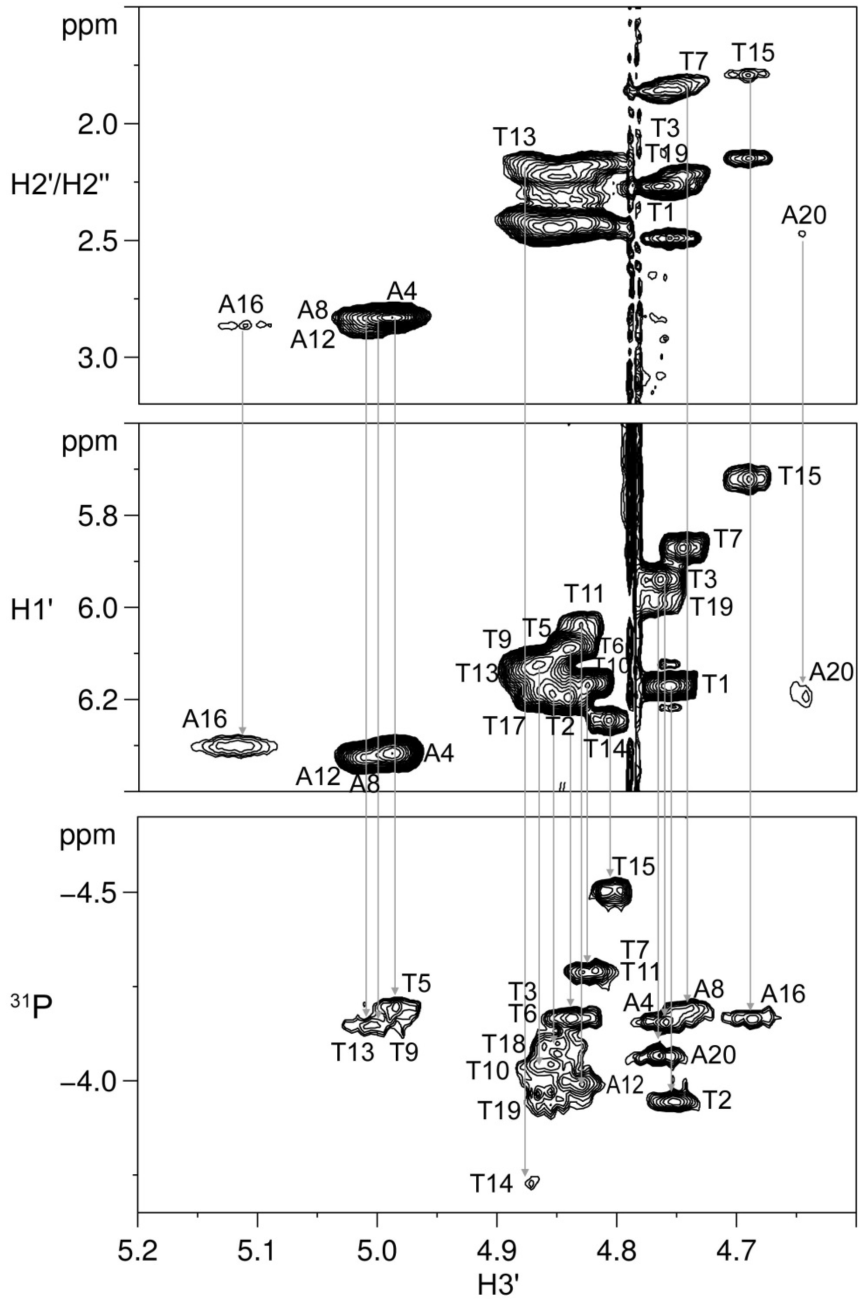

S7. ^{31}P assignments of **(a)** *(TTTA)$_6$*, **(b)** *(TTTA)$_7$*, and **(c)** *(TTTA)$_8$* using H1'-H3' TOCSY cross peaks and H3'-^{31}P HSQC cross peaks. The ^{31}P assignments could only be partially completed due to serious peak overlap. All the spectra were acquired at 25 °C.

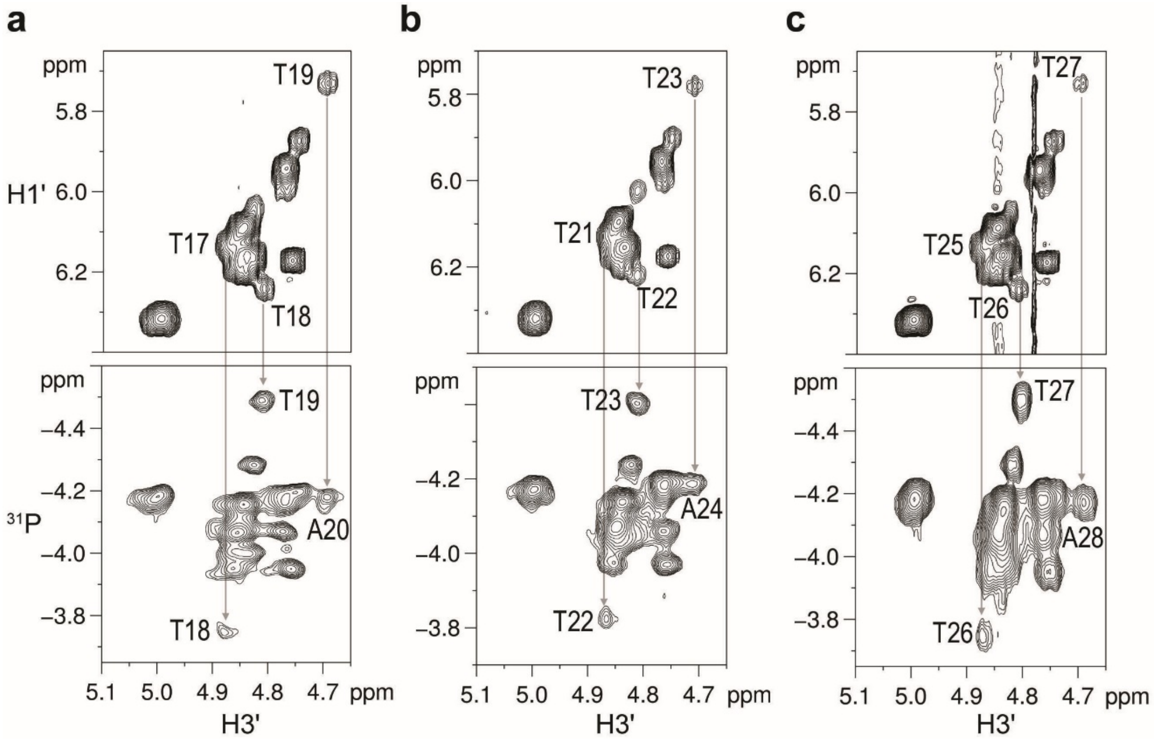

S8. ^{31}P assignments of **(a)** *A(TTTA)$_2$* at 30 °C and **(b)** *(TTTA)$_2$T* at 15 °C using H2'/H2''-H3' and H1'-H3' TOCSY cross peaks and H3'-^{31}P HSQC cross peaks.

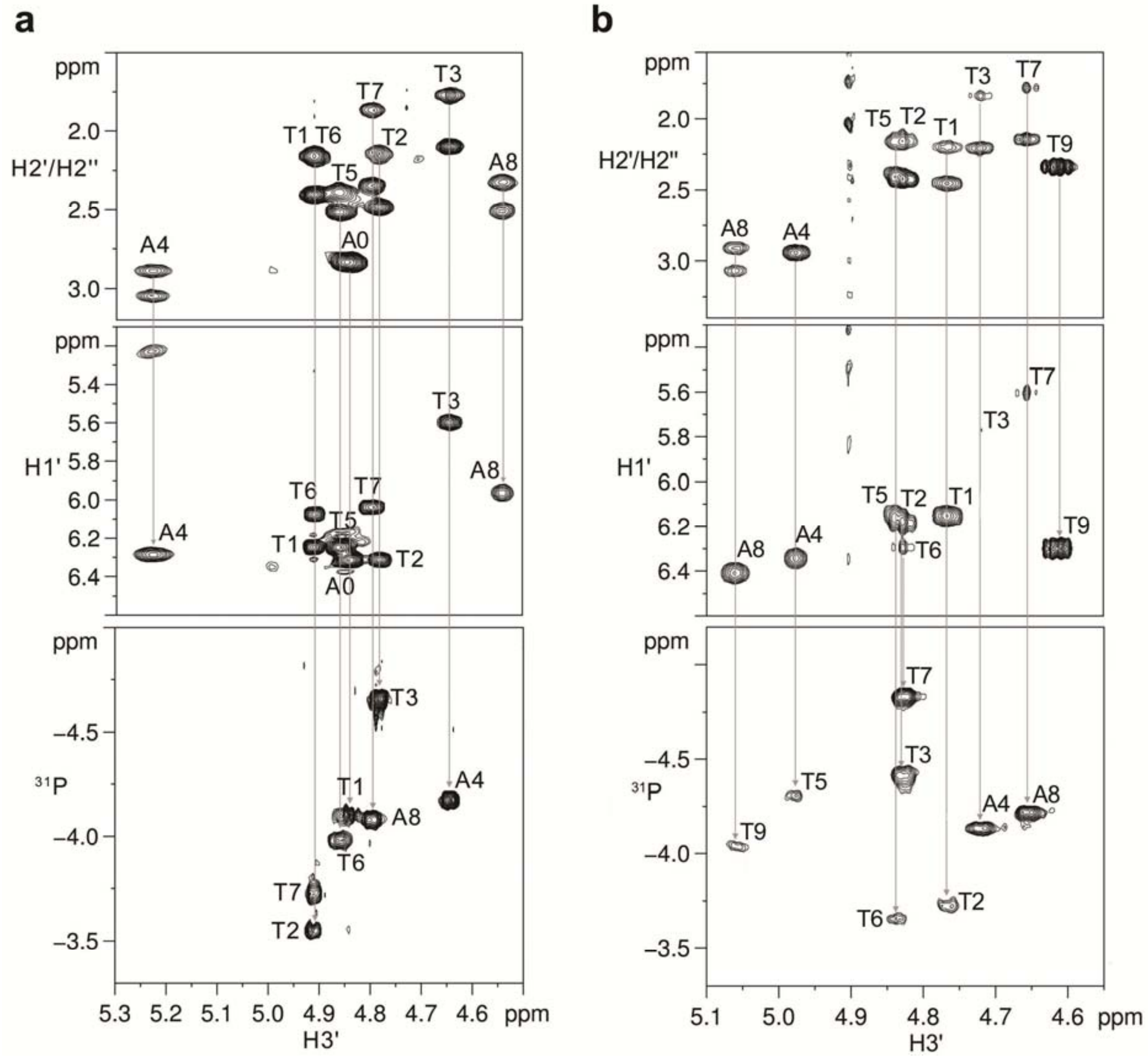

S9. Variable-temperature 1D ^{31}P spectra of *(TTTA)*$_{5\text{-}8}$ show peak broadenings in all residues at lower temperatures.

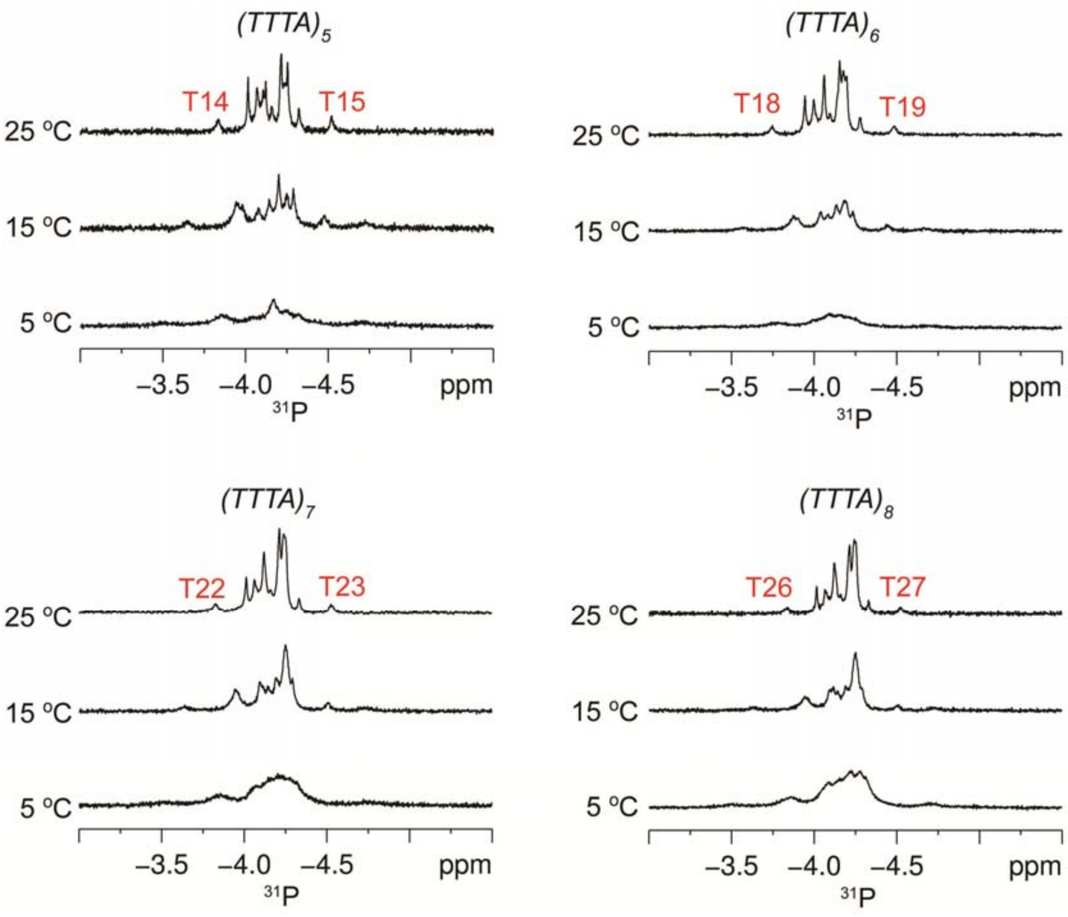

S10. The native gel results of *(TTTA)*$_{4\text{-}8}$ at **(a)** ~25 °C and **(b)** ~5 °C.

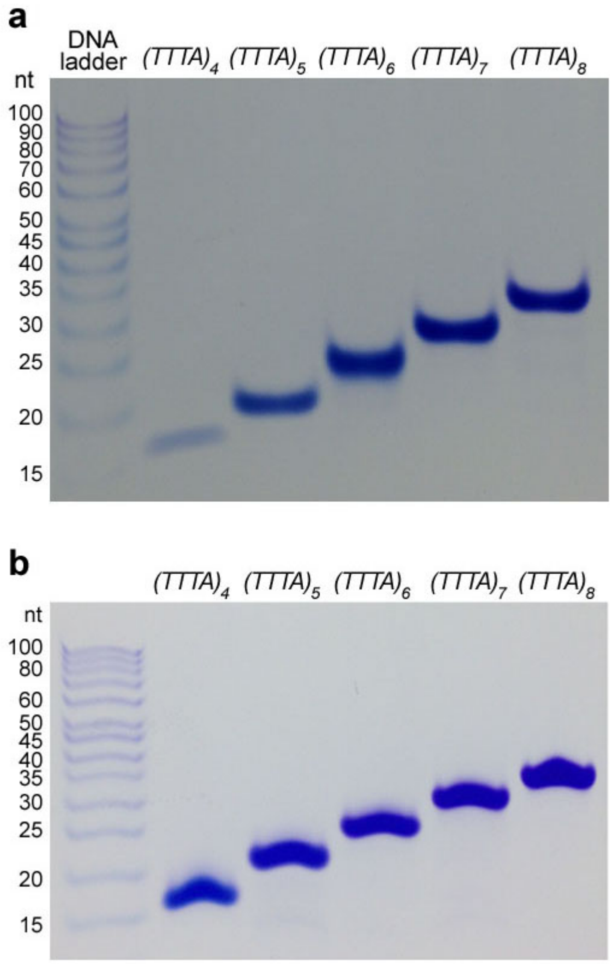

Supplement: Supplementary Figures [file sigtrans201628-s1.pdf]
